# Medical symptoms and conditions in autistic women

**DOI:** 10.1177/13623613211022091

**Published:** 2021-06-29

**Authors:** Tslil Simantov, Alexa Pohl, Alexandros Tsompanidis, Elizabeth Weir, Michael V Lombardo, Amber Ruigrok, Paula Smith, Carrie Allison, Simon Baron-Cohen, Florina Uzefovsky

**Affiliations:** 1 Ben-Gurion University of the Negev, Israel; 2 University of Cambridge, UK; 3 Istituto Italiano di Tecnologia, Italy

**Keywords:** autism, clinical, females, puberty, steroids, testosterone

## Abstract

The sex bias in autism diagnosis suggests the involvement of sex-specific endocrine mechanisms during prenatal development, but these hormones affect health throughout life. Therefore, the current study examined the association of autism and autistic traits with conditions and symptoms related to the sex-steroid system in adult women. In total, 1230 women (361 autistic), aged 15–77 years, reported on autistic traits and medical history. Medical diagnoses and symptoms were grouped by unsupervised factor analysis, and associations with autism diagnosis and autistic traits were explored. Higher rates of reproductive system diagnoses (odds ratio = 1.035, p = 0.024), prediabetes symptoms (odds ratio = 1.319, p = 0.001), irregular puberty onset (odds ratio = 1.458, p = 0.009), and menstrual length (odds ratio = 1.368, p = 0.034) and lower rates of metabolic and vascular conditions (odds ratio = 0.654, p = 0.013) were associated with diagnosis. Reproductive system diagnoses (β = 0.114, p = 0.000), prediabetes symptoms (β = 0.188, p = 0.000), menstrual length (β = 0.071, p = 0.014), irregular puberty onset (β = 0.149, p = 0.000), excessive menstruation symptoms (β = 0.097, p = 0.003), and hyperandrogenism symptoms (β = 0.062, p = 0.040) were also associated with autistic traits. Many of the conditions and symptoms found to be associated with autism or autistic traits are also related to conditions of steroid hormones and, specifically, the sex-steroid system. The study suggests an important role for steroids in autistic women, beyond prenatal development. Clinical implications are discussed.

**Lay abstract:**

Sex-steroids, such as testosterone, are thought to be one of the biological factors implicated in autism. This relies on the sex bias in the diagnosis of autism (boys are approximately four times more likely to be diagnosed than girls) and findings of associations with fetal testosterone levels in traits and abilities related to autism. The current study aimed to examine the association between medical conditions and physical symptoms, which tend to manifest in adulthood, and autism in females. Moreover, we examined their association with autistic traits throughout the spectrum. We focused on autistic women because there is little research focusing on the healthcare needs of autistic women, but those that exist suggest heightened vulnerability, and lower access to medical care. We find that conditions related to steroid hormones function are more frequent in autistic women and that they correlate with autistic traits. Specifically, we found that body mass index, reproductive system diagnoses, prediabetes symptoms, irregular puberty onset, and menstrual irregularities were significantly more frequent in autistic women and were significantly correlated with autistic traits in neurotypical women. The findings have important implications for raising awareness in autistic women of the possibility of medical conditions which might need medical attention. In addition, healthcare providers should consider these associations when performing healthcare maintenance checks and/or screening for autism.

## Introduction

Autism is a heritable condition (Gaugler et al., 2014), with genetic and biological factors consistently implicated in autism etiology (Kim & Leventhal, 2015; Lai et al., 2013). Importantly, autism confers higher risk for a series of physical symptoms and clinical co-occurring conditions (Ingudomnukul et al., 2007; Weir et al., 2021). Specifically, conditions, such as polycystic ovary syndrome (PCOS), menstrual cycle irregularity, and acne, have been previously associated with autism, indicating sex-steroid system dysfunction (Ingudomnukul et al., 2007; Pohl et al., 2014). Understanding the connection between autism and the sex-steroid system may give insight into the biological underpinnings of autism and may have clinical implications for services that autistic people need. This is particularly important in the case of autistic women, who remain an understudied (Watkins et al., 2014), yet a particularly vulnerable population with an increased risk of premature mortality (Hirvikoski et al., 2016; Hwang et al., 2019; Woolfenden et al., 2012). Indeed, autistic women show a higher load of chronic physical and mental health comorbidities, which may contribute to this mortality gap between autistic and neurotypical individuals (Croen et al., 2015; Davignon et al., 2018; DaWalt et al., 2019; Fortuna et al., 2016; Hand et al., 2019; Hwang et al., 2019; Rydzewska et al., 2018; Weir et al., 2021). Of specific interest is the increased risk for conditions related to a sex hormone imbalance (Ingudomnukul et al., 2007; Pohl et al., 2014) and wider metabolic dysfunction in autistic women (e.g. obesity, epilepsy, autoimmune disorders; (Croen et al., 2015; Davignon et al., 2018; Hand et al., 2019; Shedlock et al., 2016; Weir et al., 2021). Therefore, in the current study, we focused on examining the relationships between autism and a broad range of clinical conditions and subclinical symptoms in women, grouped into factors using exploratory factor analyses. We further investigated these associations with respect to autistic traits across the spectrum, to better understand the relevance of these findings to the broad autism phenotype (BAP; Le Couteur et al., 1996). The BAP refers to subclinical phenotypes of core symptoms of autism (i.e. social and communication impairments and repetitive behaviors), yet are subtle and do not reach the autism diagnosis threshold (Piven et al., 1997). Biological correlates of autism have also been described in relation to the BAP (Bolton et al., 1994; Ingudomnukul et al., 2007). Therefore, understanding the physical and biological correlates of clinically diagnosed autism together with BAP is of importance to better understand their role in autistic traits.

Autism has a skewed and consistent sex ratio, with males being typically diagnosed at a ratio of 4:1 (Baio et al., 2018; but see more conservative estimates of 3:1 and 2:1, respectively; Loomes et al., 2017; Mattila et al., 2011). In the last decade, it has become apparent that autistic women might have been under-recognized due to different behavioral characteristics, ascertainment bias, and bias in the diagnostic instruments (Lai et al., 2011, 2014; Whitlock et al., 2020). Yet even after considering these biases in diagnosis, it still appears that more males are diagnosed with autism (Lai et al., 2011). Higher levels of prenatal sex-steroid hormone exposure have been proposed to explain sex differences in diagnostic liability and the observed male-type shifts in specific cognitive traits in autism, compared to non-autistic men and women (Baron-Cohen, 2002; Greenberg et al., 2018).

Prenatal testosterone exposure affects brain development in humans, especially between 8 and 24 weeks of gestation (Hines, 2005). In a neurotypical population, higher levels of prenatal testosterone, as measured in amniotic fluid, have been shown to be inversely correlated with the amount of eye contact displayed at 12 months of age (Lutchmaya et al., 2002), vocabulary size at 18 and 24 months (Lutchmaya et al., 2001), and the quality of social relationships at 14 years (Lutchmaya et al., 2004). In addition, higher levels of prenatal sex-steroids were also related to autistic traits in infants and children, as measured by questionnaires validated in independent autistic cohorts (Auyeung et al., 2009). It is important to note that other studies have not found a link between prenatal or postnatal testosterone and autistic traits in the general population (Jamnadass et al., 2015; Kung, Constantinescu, et al., 2016; Kung, Spencer, et al., 2016; Tan et al., 2018; Whitehouse et al., 2012), although only one group was able to replicate the first studies by measuring testosterone directly in amniotic fluid (Kung, Spencer, et al., 2016).

Several other studies and meta-analyses have been published further indicating a link between autism likelihood and a wider endocrine dysfunction. Mothers of autistic children have been shown to have higher rates of gestational diabetes, and PCOS, in many independent cohorts around the world, which included both male and female cases (Cesta et al., 2020; Chen et al., 2020; Cherskov et al., 2018; Katsigianni et al., 2019; Rotem et al., 2021). These conditions, and particularly PCOS, have been associated with significant hormonal disruptions, leading, for example, to increased production of androgens from the placenta (Maliqueo et al., 2013) and thus supporting the possibility of a link between sex-steroids and autism in male and female fetuses (see Table 1 in the Supplementary Materials).

Aberrant hormonal exposure in utero can have lifelong effects on the endocrine health of the fetus. For example, exposure to high levels of androgens prenatally can affect the differentiation of the developing gonads and lead to PCOS in the daughters of mothers with the same syndrome (Risal et al., 2019). In autism, several studies show higher levels of circulating testosterone postnatally in childhood or adulthood, although the clinical reason for this remains unclear (e.g. Bejerot et al., 2012; Majewska et al., 2014; Ruta et al., 2011). This is also corroborated by higher rates of co-occurring symptoms related to atypical levels of androgens in autistic women (e.g. amenorrhea or severe acne) (Pohl et al., 2014).

Based on these findings, we expected to find some dysregulation of the sex-steroid system also in females, albeit with potentially different clinical presentations. The aim of the current study was to attempt to fill this gap in current knowledge.

We summarize the main findings regarding the relationship between autism in adulthood and conditions related to sex-steroid imbalance in Table 1. We have divided these into four main groups: (1) medical conditions, (2) subclinical symptoms, (3) timing of puberty, and (4) reproductive health. For most of the conditions presented in Table 1, a direct or indirect association with sex-hormone imbalance, and particularly testosterone, has been found. Notwithstanding this apparent link, these conditions have been examined separately, and the combined associations with autism have not been previously thoroughly explored. For more details, see Table 1 in the Supplementary Materials.

**Table 1.** The relationship between autism and medical conditions, hormonal symptoms, puberty, and reproductive health in women.

Category	Syndrome/symptom	Associated with autistic traits (T)/diagnosis (D)/no significant difference (NS)	References
Medical conditions	PCOS	T	Hergüner et al. (2012)
		D	Cherskov et al. (2018); Ingudomnukul et al. (2007); Pohl et al. (2014)
	Type I diabetes	D	Freeman et al. (2005)
	Diabetes mellitus	D	Flygare Wallén et al. (2018); Hand et al. (2019)
		D—males only	Weir et al. (2021)
	Diabetes (not specific)	NS	Ingudomnukul et al. (2007)
	Maternal type II diabetes	D	Xiang et al. (2015)
	Prediabetes	D—females only	Weir et al. (2021)
	Obesity	D	Croen et al. (2015); Davignon et al. (2018); Hand et al. (2019); Zheng et al. (2017)
	Hypertension	D	Croen et al. (2015); Hand et al. (2019); Weiss et al. (2018)
	Dyslipidemia/lipid metabolism disorders	D	Croen et al. (2015); Davignon et al. (2018); Hand et al. (2019); Sikora et al. (2006); Tierney et al. (2001); Vohra et al. (2017)
	Epilepsy	D	Croen et al. (2015); Davignon et al. (2018); Hand et al. (2019); Ingudomnukul et al. (2007); Pohl et al. (2014); Vohra et al. (2017)
	Cancers (particularly hormone-associated)	D	Chiang et al. (2015); Hand et al. (2019); Kao et al. (2010)
		NS	Ingudomnukul et al. (2007)
	Cancer (not particular)	NS	Weir et al. (2021)
	Autoimmune disorders	D	Croen et al. (2015); Davignon et al. (2018)
	Thyroid disorders	D	Croen et al. (2015); Davignon et al. (2018); Hand et al. (2019); Vohra et al. (2017)
		NS	Ingudomnukul et al. (2007)
	CAH	T	Knickmeyer, Baron-Cohen, et al., 2006
		NS	Ingudomnukul et al. (2007)
	Cholesterol imbalance	D	Sikora et al. (2006); Tierney et al. (2001)
		NS—females only	Weir et al. (2021)
	High blood pressure	NS	Weir et al. (2021)
	Low blood pressure	D—females only	Weir et al. (2021)
	PMS	NS	Ingudomnukul et al. (2007)
	Cardiac arrhythmia, atrial fibrillation, or other cardiac conditions	D	Weir et al. (2021)
		NS	Ingudomnukul et al. (2007)
Hormone-related symptoms	Hirsutism	D	Ingudomnukul et al. (2007)
	Severe acne	D	Ingudomnukul et al. (2007); Pohl et al. (2014)
	Dysmenorrhea (severe menstrual cramps)	D	Ingudomnukul et al. (2007); Pohl et al. (2014)
	Excessive menstrual bleeding or endometriosis	NS	Ingudomnukul et al. (2007)
Puberty	Precocious puberty	D	Corbett et al. (2020); Mouridsen & Larsen (1989); Yoshimura et al. (2005)
		NS	Ingudomnukul et al. (2007)
	Delayed puberty	D	Knickmeyer, Wheelwright, et al. (2006)
		T	Whitehouse et al. (2011)
	Typical pubertal timing	D	May et al. (2017)
Reproductive health	Irregular menstrual cycle	D	Ingudomnukul et al. (2007)
	Amenorrhea	D	Pohl et al. (2014)

PCOS: polycystic ovary syndrome; CAH: congenital adrenal hyperplasia; PMS: premenstrual syndrome.

Past studies tended to examine each condition/group of conditions separately. Yet, many of these conditions tend to co-occur (Pasquali & Pignatelli, 2019); to pinpoint the specific associations, the conditions must be analyzed concurrently. Therefore, the current study aimed to comprehensively examine the association between sex-steroid conditions and autism in women. This allows to better characterize the relative importance of each condition, while controlling for others. We conducted two main studies. In Study 1, we examined the relative frequency of steroid-related conditions, symptoms, atypical onset of puberty, and reproductive health conditions in adult women with and without autism. We hypothesized that higher rates of these would be related to autism. In Study 2, we examined these conditions in association with autistic traits across the population.

## Methods

### Participants

The study included N = 1230 women, aged 15–77 (M = 38.42, SD = 12.4) years. Of those, N = 361 autistic women (see Table 2 for details of sample composition). Women were included based on their biological sex only. Diagnostic status is based on participants’ reports, including specific details regarding their diagnosis (e.g. date of diagnosis). Importantly, some women reported having no official diagnosis, but did suspect that they were autistic (N = 117). As there was no official diagnosis, these women were included in the non-autistic group in the main analyses. To examine the effect of this decision, another set of analyses was conducted without this group. Information regarding participants’ racial and ethnic background was not collected.

**Table 2.** Sample demographics.

	Women with an autism diagnosis (N = 361)			Women without an autism diagnosis (N = 869)		
	M	SD	Range	M	SD	Range
Age (years)	38.42	12.4	15.39–73.42	42.33	11.44	15.07–77.4
BMI	26.46	7.07	13.63–70.03	26.25	6.44	15.24–79.08
Family history of autism	N = 85 (23.55%)			N = 139 (16.01%)		

SD: standard deviation; BMI: body mass index.

Participants took part in the study through two websites, managed by the Autism Research Centre (ARC) at the University of Cambridge, UK. The first website (https://autismresearchcentre.co.uk/) is targeted at autistic individuals and their family members. The second website (https://cambridgepsychology.com) is targeted at the general population and the specific affiliation to “autism” and the ARC is not mentioned. The study was advertised as a “health and pregnancy questionnaire,” available for anyone registered on either of the two websites. Participants were provided with information regarding the study and gave their consent before accessing the questionnaire. The study was approved by the Psychology Research Ethical Committee (PREC) at Cambridge University.

### Measures

Participants completed the following questionnaires online: a demographic questionnaire where participants reported on their date of birth and birth sex, the Autism Spectrum Quotient (AQ), and a self-report questionnaire, assessing autistic traits (Baron-Cohen et al., 2001). The questionnaire contains 50 items, each of them scores 1 if the respondent records the autistic-like behavior either mildly or strongly.

The health and pregnancy questionnaire. This questionnaire was developed with the help of focus groups in which participated women either diagnosed with autism or mothers of autistic children. Within the focus groups, women talked about the medical conditions and symptoms which most affect them. Based on the collected information, the questionnaire elicited information regarding endocrine-related (and especially testosterone-related) conditions and symptomatology, and physical health (see the full questionnaire in the Supplementary Materials). Topics covered in the questionnaire are as follows: (a) Onset and characteristics of puberty. Participants were asked to rate the timing of their symptom appearance (breasts, body hair, facial hair, voice, and growth spurt) on a scale of (−2) “much earlier than,” (−1) “a little earlier,” (0) “about the same time,” (+1) “a little later,” and (+2) “much later than peers.” Items were coded in binary form: 0 for typical and 1 for atypical onset—either early or late appearance of puberty symptoms. This dichotomization was chosen based on previous studies reporting both early and late puberty in autism (Knickmeyer, Wheelwright, et al., 2006; Mouridsen & Larsen, 1989; Tordjman et al., 1997; Yoshimura et al., 2005). The questions are informed by the Tanner (1962) stages of puberty; (b) Reproductive health, particularly regarding menstrual cycle, including age of menarche, length, consistency, and related symptoms of the menstrual cycle; (c) Medical conditions related to hormonal imbalance, including congenital adrenal hyperplasia (CAH), type I diabetes, type II diabetes, hypogonadism, hyperthyroidism, hypothyroidism, precocious puberty, delayed puberty, autoimmune disorder, breast cancer, cardiac conditions, epilepsy, hypertension, and high cholesterol. Other diagnoses enquired about were PCOS, ovarian cancer, uterine cancer, anovulation, and premenstrual syndrome (PMS). The questionnaire also included chronic fatigue syndrome (CFS), myalgic encephalomyelitis (ME), and post-viral fatigue syndrome (PVFS) in a single item, and therefore, we used a single variable to represent these; (d) Physical symptoms related to sex-steroid imbalance and undiagnosed diabetes, including extreme thirst (undiagnosed diabetes symptom), frequent need to urinate (undiagnosed diabetes symptom), hair loss (sex-steroid imbalance symptom), severe acne (sex-steroid imbalance symptom), and sudden weight loss (both undiagnosed diabetes and sex-steroid imbalance symptom). Additional sex-specific symptoms were also examined: excessive body or facial hair, excessive menstrual bleeding, and unusually painful periods; (e) Physical health as reflected in current weight and height.

## Data analysis

The study examined the association between conditions and symptoms of hormonal imbalance and (1) a diagnosis of autism and (2) autistic traits.

### Data reduction

Hormonal imbalance was captured through nine variables, based on the following characteristics of clinical history and related symptoms:

1. BMI—participants reported their height and weight, and their current BMI was calculated. The current BMI was used in the analyses.
2. Puberty onset and symptoms (Puberty, above)—the four items pertaining to the timing of puberty onset were averaged to create a puberty onset score.
3. Length of menstrual cycle (Reproductive health, above)—the answers to the question were coded in binary form—0 denotes typical length of menstrual cycle (25–34 days), 1 denotes atypical length (less than 25 days or more than 34 days).
4. Consistency of the menstrual cycle (Reproductive health, above)—the answers to the question were coded in a similar way—0 denotes a very consistent (“highly consistent”) and 1 denotes less consistent menstrual cycle (“fairly consistent”, “fairly variable”, “highly variable”).
5. Hormonal disorders (Medical diagnoses, above)—the questionnaire probes for the existence of 20 medical diagnoses related to hormonal function, resulting in 20 binary responses. Information regarding their frequency in the sample can be found in Supplementary Table 2. Of the 20 conditions, 5 were removed from further analyses based on very low frequency in the current sample: hypogonadism (0.2%); type I diabetes (0.1%); precocious puberty (0.5%); delayed puberty (0.4%); and CAH (0%). Following removal of these, 15 diagnoses were used in the analyses. To reduce the number of variables, a confirmatory categorical principal component analysis (CATPCA) was conducted. The CATPCA is used to group related binary variables into factors. A three-factor model was selected based on both fit indices and the descriptive power of each factor. See Supplementary Table 3 for details. The factors that emerged relate to (1) metabolic and vascular health (including high blood pressure, high cholesterol, and type II diabetes); (2) immunity-related diagnoses (including autoimmune disorder, hyperthyroidism, and hypothyroidism); and (3) reproductive system diagnoses (including anovulation, PCOS, PMS, ovarian cancer, and uterine cancer). Count scores for each of the factors (number of items endorsed as “yes”) were calculated and used in further analyses.

6. Hormonal symptoms (Physical symptoms, above)—the questionnaire probes for various symptoms related to hormonal imbalance, under the assumption that some symptoms exist but do not necessarily reach a clinical diagnostic threshold or have not been brought to the attention of a physician. Confirmatory CATPCA was again used for data reduction of the hormonal symptoms data. A three-factor model was selected based on both fit indices and the descriptive power of each factor. See Supplementary Table 7 for details. The factors that emerged are (1) prediabetes symptoms (including extreme thirst, frequent need to urinate, hair loss or thinning, and sudden, unexplained weight loss); (2) excessive menstruation symptoms (including unusually painful periods and excessive menstrual bleeding); and (3) hyperandrogenism symptoms (including hirsutism and severe acne). Again, a count score for each factor was computed and used in further analyses.

Missing values—the variables BMI (6.2% missing), puberty onset (9.02% missing), and consistency of the menstrual cycle (12.8% missing) had missing values, which were replaced by their respective mean scores.

To summarize, the above-described items were grouped into nine variables which were used in all analyses: metabolic and vascular health, reproductive system diagnoses, immunity-related diagnoses, prediabetes symptoms, excessive menstruation symptoms, hyperandrogenism symptoms, irregular puberty onset, menstrual length, and menstrual consistency.

### Analysis

Two separate analyses were conducted to examine the relationship between these conditions: (1) autism diagnosis and (2) autistic traits. For Study 1, a binomial logistic regression with autism diagnosis as the dependent variable (DV) was executed. The predictor variables were as described above. Age and BMI were controlled for in all analyses.

In Study 2, a hierarchical regression was performed using AQ score (Baron-Cohen et al., 2001) as the DV. Age and BMI were controlled for in all analyses. Three of the six count variables were dummy-coded (metabolic and vascular health, excessive menstruation symptoms, and hyperandrogenism symptoms). The other three count variables (prediabetes symptoms, reproductive system diagnoses, and immunity-related diagnoses), at least one of the dummy variables, had a frequency of zero (e.g. some people endorsed having zero to one condition, and some having three to four conditions, but none reported having two conditions) and therefore could not be examined as a dummy variable. For these variables, the count score was examined in future analyses.

Community involvement. The questionnaire used in this study was developed based on the input provided by autistic women and mothers of autistic children, using several focus groups, led by A.P. Focus groups were held to investigate the most prevalent medical conditions in autistic women, and the health and pregnancy questionnaire was developed accordingly. We sincerely thank the women who participated in these groups and helped pinpoint the specific medical and physiological concerns that autistic women face.

## Results

**Figure 1.**
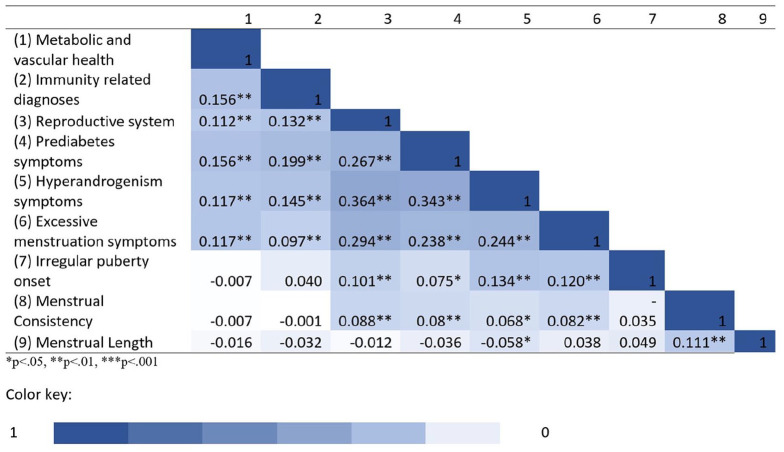
A heat map of correlations between the predictors.

Figure 1 presents the correlation structure between the components examined in the following analyses. All factors correlated with at least one other factor, and most are correlated with multiple factors. This exemplifies the necessity to examine all factors jointly in relation to autism, as a way of controlling for the shared variance among them.

### Study 1—predicting autism diagnosis

A logistic regression model was used to predict autism diagnosis from the nine predictors. The model was statistically significant, χ^2^(11) = 87.190, p = 0.0000, explaining 10.8% (Nagelkerke R^2^) of the variance in autism diagnosis and correctly classifying 72.2% of cases (sensitivity = 13.8%, specificity = 95.8%). Of the nine predictors, five were statistically significant: metabolic and vascular health, reproductive system diagnoses, prediabetes symptoms, irregular puberty onset, and menstrual length (as shown in Table 3). Women with prediabetes symptoms, reproductive system diagnoses, or irregular menstrual length were more likely to be autistic than non-autistic women. Metabolic and vascular health was associated with a reduction in the likelihood of being autistic.

**Table 3.** Predictors of autism diagnosis among women.

	β	p-value	OR	95% CI	
				Lower	Upper
Age	−0.026***	0.000	0.974	1.014	1.039
BMI	0.004	0.706	1.004	0.976	1.017
Metabolic and vascular health	−0.425*	0.013	0.654	1.092	2.142
Reproductive system diagnoses	0.259*	0.024	1.035	0.617	0.966
Immunity-related diagnoses	0.249	0.085	0.966	0.587	1.035
Prediabetes symptoms	0.277**	0.001	1.319	0.644	0.893
Excessive menstruation symptoms	0.135	0.386	1.144	0.645	1.185
Hyperandrogenism symptoms	0.249	0.085	1.283	0.587	1.035
Irregular puberty onset	0.377**	0.009	1.458	0.516	0.911
Menstrual length	0.313*	0.034	1.368	0.547	0.977
Menstrual consistency	−0.213	0.207	0.808	0.889	1.722

CI: confidence interval; OR: odds ratio; BMI: body mass index.

* p < 0.05, **p < 0.01, ***p < 0.001.

To supplement our findings, we conducted a sensitivity analysis excluding women from the neurotypical group who suspected they may be autistic but were not clinically diagnosed. The analysis revealed very similar results (see Table 4). Women with prediabetes symptoms, reproductive system diagnoses, or irregular menstrual length were more likely to be autistic than non-autistic women. Metabolic and vascular health was again associated with a reduction in the likelihood of having an autism diagnosis. In addition, having excessive menstruation symptoms was also significantly associated with having an autism diagnosis.

**Table 4.** Predictors of autism diagnosis among women (excluding suspected autism cases).

	β	p-value	OR	95% CI	
				Lower	Upper
Age	−0.028***	0.000	0.973	1.015	1.041
BMI	0.006	0.571	1.006	0.973	1.015
Metabolic and vascular health	−0.418*	0.021	0.658	1.065	2.167
Reproductive system diagnoses	0.256*	0.033	1.292	0.612	0.980
Immunity-related diagnoses	0.203	0.175	1.225	0.608	1.095
Prediabetes symptoms	0.344**	0.000	1.411	0.592	0.848
Excessive menstruation symptoms	0.186	0.060	1.204	0.684	1.008
Hyperandrogenism symptoms	0.182	0.273	1.200	0.602	1.154
Irregular puberty onset	0.484**	0.001	1.213	0.461	0.825
Menstrual length	0.305*	0.047	1.005	0.546	0.996
Menstrual consistency	−0.141	0.415	0.618	0.820	1.618

CI: confidence interval; OR: odds ratio; BMI: body mass index.

* p < 0.05, **p < 0.01, ***p < 0.001.

### Study 2—predicting autistic traits

A hierarchical linear regression analysis was used to examine medical conditions and symptoms associated with autistic traits, as measured by the AQ (Baron-Cohen et al., 2001) in both women with and without a diagnosis of autism (N = 1230). The analysis showed that age and BMI were significantly associated with AQ scores (F(2, 999) = 19.736, p < 0.001) and accounted for 3.8% of the variance. In addition, reproductive system diagnoses, prediabetes symptoms, menstrual length, irregular puberty onset, excessive menstruation symptoms, and hyperandrogenism symptoms were positively associated with autistic traits. See details in Table 5. It is worth noting that adding reproductive system diagnoses, prediabetes symptoms, menstrual length, and irregular puberty onset (Step 2) accounted for most of the variance, suggesting a larger role than excessive menstruation symptoms in predicting autistic traits.

**Table 5.** Summary of hierarchical regression analysis predicting AQ scores among women.

Variable	β	t	R	R^2^	ΔR^2^	p-value
Step 1			0.189	0.036	0.036	0.000
Age	−0.178***	−5.884				0.000
BMI	0.090**	2.973				0.003
Step 2			0.368	0.135	0.099	0.000
Reproductive system diagnoses	0.114***	3.783				0.000
Immunity-related diagnoses	0.035	1.203				0.229
Prediabetes symptoms	0.188***	6.296				0.000
Menstrual length	0.071*	2.474				0.014
Menstrual consistency	0.013	0.447				0.655
Irregular puberty onset	0.149***	5.174				0.000
Step 3			0.378	0.143	0.008	0.009
Excessive menstruation—one symptom	0.052	1.716				0.087
Excessive menstruation—two symptoms	0.097**	2.969				0.003
Step 4			0.385	0.148	0.005	0.044
Hyperandrogenism symptoms—one symptom	0.062*	2.057				0.040
Hyperandrogenism symptoms—two symptoms	0.049	1.679				0.093
Step 5			0.387	0.150	0.002	0.537
Metabolic and vascular health—one syndrome	−0.026	−0.904				0.366
Metabolic and vascular health—two syndromes	0.005	0.176				0.861
Metabolic and vascular health—three syndromes	0.032	1.068				0.286

AQ: Autism Spectrum Quotient; BMI: body mass index.

* p < 0.05, **p < 0.01, ***p < 0.001.

### Predicting autistic traits among non-autistic women

A second hierarchical linear regression analysis was conducted to ascertain the relevance of the findings for women without autism. The analysis revealed similar findings. Age and BMI were significantly associated with AQ scores (F(2, 717) = 8.800, p < 0.001) and accounted for 2.3% of the variance. The predictors’ reproductive system diagnoses, prediabetes symptoms, and irregular puberty onset were positively associated with autistic traits. The other predictors showed no significant association with AQ scores (Table 6). Similar to the previous analysis, reproductive system diagnoses, prediabetes symptoms, and irregular puberty onset (Step 2) accounted for most of the variance in predicting autistic traits. The similarity of these results to those of the combined sample suggests that the general findings were not driven by the autism group, and similar effects can be observed in the neurotypical population.

**Table 6.** Summary of hierarchical regression analysis predicting AQ scores for the comparison group only.

Variable	β	t	R	R^2^	ΔR^2^	p-value
Step 1			0.153	0.023	0.023	0.000
Age	−0.134***	−3.747				0.000
BMI	0.091*	2.531				0.012
Step 2			0.321	0.103	0.080	0.000
Reproductive system diagnoses	0.117**	3.263				0.001
Immunity-related diagnoses	0.018	0.498				0.619
Prediabetes symptoms	0.174***	4.927				0.000
Menstrual length	0.053	1.547				0.122
Menstrual consistency	0.012	0.350				0.727
Irregular puberty onset	0.138***	3.990				0.000
Step 3			0.333	0.111	0.008	0.040
Excessive menstruation—one symptom	0.061	1.706				0.088
Excessive menstruation—two symptoms	0.092*	2.300				0.022
Step 4			0.338	0.114	0.003	0.230
Hyperandrogenism symptoms—one symptom	0.052	1.424				0.155
Hyperandrogenism symptoms—two symptoms	0.040	1.122				0.262
Step 5			0.342	0.117	0.003	0.486
Metabolic and vascular health—one syndrome	0.027	0.758				0.449
Metabolic and vascular health—two syndromes	0.031	0.892				0.373
Metabolic and vascular health—three syndromes	0.045	1.224				0.221

AQ: Autism Spectrum Quotient; BMI: body mass index.

* p < 0.05, **p < 0.01, ***p < 0.001.

We repeated the analysis after excluding women who suspected they might be autistic but did not have a clinical diagnosis (N = 738, mean AQ score = 18.69). The analysis revealed similar findings. Age and BMI were significantly associated with AQ scores (F(2, 673) = 10.000, p < 0.001) and accounted for 2.9% of the variation. Reproductive system diagnoses, prediabetes symptoms, and irregular puberty onset were positively associated with autistic traits. In addition, having one symptom of excessive menstruation was also significantly associated with autistic traits. The other predictors showed no significant association with autistic traits. Similarly to the previous analyses, reproductive system diagnoses, prediabetes symptoms, and irregular puberty onset (Step 2) accounted for most of the variance than hyperandrogenism symptoms in predicting autistic traits. See details in Table 7.

**Table 7.** Summary of hierarchical regression analysis predicting AQ scores for the comparison group only (excluding suspected autism cases).

Variable	β	t	R	R^2^	ΔR^2^	p-value
Step 1			0.170	0.029	0.029	0.000
Age	−0.153***	−3.98				0.000
BMI	0.097*	2.518				0.012
Step 2			0.289	0.083	0.054	0.000
Reproductive system diagnoses	0.104**	2.672				0.008
Immunity-related diagnoses	0.028	0.706				0.480
Prediabetes symptoms	0.153***	3.926				0.000
Menstrual length	0.045	1.205				0.229
Menstrual consistency	−0.027	−0.725				0.469
Irregular puberty onset	0.086*	2.280				0.023
Step 3			0.297	0.088	0.005	0.176
Excessive menstruation—one symptom	0.059	1.494				0.136
Excessive menstruation—two symptoms	0.065	1.493				0.136
Step 4			0.309	0.096	0.008	0.063
Hyperandrogenism symptoms—one symptom	0.084*	2.124				0.034
Hyperandrogenism symptoms—two symptoms	0.048	1.251				0.211
Step 5			0.310	0.096	0.000	0.953
Metabolic and vascular health—one syndrome	−0.002	−0.044				0.965
Metabolic and vascular health—two syndromes	−0.005	−0.117				0.907
Metabolic and vascular health—three syndromes	0.021	0.528				0.597

AQ: Autism Spectrum Quotient; BMI: body mass index.

* p < 0.05, **p < 0.01, ***p < 0.001.

## Discussion

The current study aimed to examine the relationship between autism or autistic traits in women and a host of conditions related to steroid hormones function. Our hypotheses that these are more frequent in autistic women and that they correlate with autistic traits were largely confirmed. Specifically, we found that BMI, reproductive system diagnoses, prediabetes symptoms, irregular puberty onset, and menstrual irregularities were significantly more frequent in autistic women. In addition, these factors were significantly correlated with autistic traits in neurotypical women. Finally, metabolic and vascular health was significantly negatively associated with women having an autism diagnosis after controlling for age and BMI, but not with autistic traits. Taken together, along with previous findings regarding the connection between testosterone and medical conditions, hormonal symptoms, puberty, and reproductive health (see Table 1 in Supplementary Materials), the findings suggest that autism in women may be part of a wider syndrome of endocrine dysfunction that specifically includes imbalances in the sex-steroid system. Also, reproductive system diagnoses, prediabetes symptoms, menstrual length, and irregular puberty onset were more predictive of autistic traits than excessive menstruation symptoms.

*Reproductive system diagnoses* (PCOS, PMS, anovulation, ovarian cancer, and uterine cancer) were found to be significant predictors of both having an autism diagnosis and autistic traits in this study’s population of women, when examined as a group. This finding is in accordance with previous studies reporting higher rates of PCOS, ovarian cancer, genitourinary cancer, and family history of uterine neoplasms (benign or cancerous) in autism (Cherskov et al., 2018; Chiang et al., 2015; Hergüner et al., 2012; Ingudomnukul et al., 2007; Pohl et al., 2014). Although the common etiology for this diverse group of conditions is unclear, it most likely involves an interplay between genetic susceptibility and sex-steroid signaling. For example, elevated androgen levels and estrogenic signaling have both been associated with greater risk of endometrial cancer (Kaaks et al., 2002; reviewed in Rodriguez et al., 2019). Particularly for PCOS, exposure to excess androgens in fetal life has been shown to affect the developing gonads and induce the condition in both animal models and humans (Risal et al., 2019). PCOS also includes both elevated levels of androgens and estrogens, and more frequent occurrence of neoplasms of the reproductive system (Maliqueo et al., 2013; Yin et al., 2019). Interestingly, PCOS also relates to endocrine health beyond sex-steroids, with lifetime metabolic effects, including increased adiposity, increased BMI, and insulin resistance. The implication of high BMI and prediabetes in this study confirms previous findings (Croen et al., 2015; Davignon et al., 2018; Hand et al., 2019; Weir et al., 2021; Zheng et al., 2017) and could potentially be traced back to prenatal sex-steroids exposure as well; it may represent evidence of metabolic dysfunction in autistic women that is similar to the wider endocrine features of PCOS.

Prediabetic symptoms, such as excessive thirst and frequent urination, could be considered as signs of insulin resistance, as is often the case in the metabolic syndromes of obesity and PCOS, rather than idiopathic type II diabetes. This is also consistent with previous findings that indicate that autistic females are at increased risk of prediabetes but not type II diabetes (Weir et al., 2021). An alternative explanation to the findings may relate to the effects of other steroids hormones, such as aldosterone or autonomic nervous system dysfunction, which have been previously reported in autistic individuals (Kushki et al., 2014).

Results showed that autism is a predictor of higher rates of prediabetes symptoms but lower rates of metabolic and vascular disease (including high blood pressure, high cholesterol, and type II diabetes). This pattern may reflect disparities in healthcare access and pre-symptomatic testing between autistic and neurotypical women: hypertension, high cholesterol, and type II diabetes require medical and laboratory tests, and many patients are admitted to the hospital with uncontrolled, undiagnosed diabetes with end-stage complications without previously knowing that they are diabetic (Craig, 1999; Heller & Marks, 2002). It is possible that autistic women are less likely to access healthcare than neurotypical women, given socialization difficulties, which could explain the lower rates of these conditions. Therefore, this specific difference may reflect sociological and not an underlying physiological difference (Nicolaidis et al., 2013). Alternatively, autistic people may have different mediators of metabolic disease, compared to the neurotypical population. Several clinical cohort studies show deficiencies in cholesterol metabolism in autism, within and outside sex-steroid pathways (Sikora et al., 2006; Tierney et al., 2001). More research would be needed to confirm whether prediabetes and related conditions in autism are independent to cholesterol changes and whether alternative screening tests are warranted in this population.

Higher rates of hypertension in autistic women could also be related to diabetes-related vascular changes (Anderson & Rocchini, 1993), aldosterone (Gaddam et al., 2008; Vasan et al., 2004), and/or the autonomic nervous system (Mancia & Grassi, 2014). They may also be related to high levels of anxiety, which are the characteristic of autistic individuals (Gillott et al., 2001; Gillott & Standen, 2007; White et al., 2009). Differences in severity and in the corresponding stress could potentially also explain why hypertension was significantly associated in the case-control comparison but did not correlate linearly to autistic traits in this study.

Atypical puberty onset was also associated both with having an autism diagnosis and with autistic traits among the recruited cohort of women. Previous clinical and epidemiological studies have also reported associations between puberty onset and autism with mixed results as to whether this relationship is due to precocious or late puberty (Corbett et al., 2020; Knickmeyer, Wheelwright, et al., 2006; Mouridsen & Larsen, 1989; Whitehouse et al., 2011; Yoshimura et al., 2005). In terms of the biological pathways, sex-steroids and aromatization of testosterone and estradiol, have been shown to be important regulators of puberty onset, by acting on kisspeptin neurons, which in turn control gonadotropin hormone-releasing hormone (GnRH) secretion (Gonzalez et al., 2014; Poling & Kauffman, 2013). This pattern is apparent in humans, as evidenced by delayed puberty onset in rare cases of aromatase deficiency in females (Belgorosky et al., 2009). The GABAergic system is also important in the effects of estradiol on the hypothalamic axis and the regulation of puberty timing (Perrot-Sinal et al., 2001). In autistic individuals, both irregular puberty timing and increased prenatal sex-steroids may thus indicate the same hypothalamic dysfunction relating to the excitatory/inhibitory ratio and GABAergic signaling in particular (Cellot & Cherubini, 2014).

The results of the current study also suggest a link between autism and menstrual characteristics, as excessive menstruation and menstrual length were significant predictors of both having an autism diagnosis and autistic traits. Similarly to insulin resistance, PMS has also been linked to metabolic dysfunction (Hashemi et al., 2016), PCOS, and higher levels of steroids, such as progesterone and estrogens (Hammarback et al., 1989), all of which have also been associated with autistic individuals (Cherskov et al., 2018; Croen et al., 2015; Davignon et al., 2018; Hand et al., 2019; Hergüner et al., 2012; Ingudomnukul et al., 2007; Pohl et al., 2014; Sikora et al., 2006; Tierney et al., 2001; Vohra et al., 2017).

In this study, autoimmune disorders were not associated with having an autism diagnosis, or with autistic traits. This finding complements previous studies suggesting that autoimmune disorders are related to autism only in individuals with a specific family history of autoimmune disorder (Andersen et al., 2014; Atladóttir et al., 2009; Brown et al., 2015; Keil et al., 2010). Larger epidemiological studies have reported a less specific association (Croen et al., 2015; Davignon et al., 2018).

Taken together, we found that in a cohort of women recruited online, autistic women had a higher load of medical conditions and physical symptoms that relate to sex-steroids function. Interestingly, many of these physiologic features are also present in the wider spectrum of autistic traits in the neurotypical population, suggesting a common continuum of liability for both endocrine health and neurodevelopment. These physiological manifestations of hormonal imbalance could thus be viewed as part of a neuroendocrine syndrome in autistic women and indirect evidence of common biological underpinnings relating to the function and regulation of sex-steroids.

## Clinical implications

The current study has several clinical implications both for autistic individuals and healthcare providers. First, the current study bolsters the evidence that women with conditions linked to sex-steroids may be more likely to have autism. Physicians should consider possible associations between sex-steroid-related health conditions and autism when performing healthcare maintenance checks and/or screening for autism. Similarly, autistic females should also be made aware of the possibility of accompanying medical conditions, allowing them to recognize potentially worrying symptoms of sex-steroid imbalance and prediabetes and to better advocate for themselves during healthcare visits. Yet, the population of autistic women has been often overlooked, with severe implications for mental and physical health (Zener, 2019), which in turn may relate to higher mortality rates (DaWalt et al., 2019; Hwang et al., 2019), and increased risk of suicidality (Cassidy et al., 2018; Hirvikoski et al., 2016), which is a major health concern in autism. Second, these findings may also have important implications for mental health practitioners, in supporting women with social communication challenges to express their concerns. Third, the contradicting finding regarding prediabetes symptoms and type II diabetes may suggest that autistic women are less likely to receive a clinical diagnosis of type II diabetes, possibly due to social communication and self-advocacy challenges. Indeed, the strength of the current study is the examination of medical symptoms that do not reach the threshold for a clinical diagnosis. Some symptoms of sex-hormone imbalance manifest in what is viewed as cosmetic symptoms (e.g. hirsutism, acne) and may be less likely to be brought to the attention of a physician or be recorded in medical records. Thus, the current study provides unique insights regarding subclinical symptoms, as current epidemiological studies mainly rely on retrospective analysis of medical records.

Studies of autistic individuals have revealed that, despite higher health care costs and utilization (Tregnago & Cheak-Zamora, 2012; Vohra et al., 2017; Weiss et al., 2018; Zerbo et al., 2019), autistic individuals have worse access to medical care, and specifically for gynecological visits and cervical cancer screenings (Tregnago & Cheak-Zamora, 2012; Zerbo et al., 2019). In addition, autistic adults report having lower satisfaction with patient-provider communication, lower levels of self-efficacy, higher odds of unmet health care needs, higher use of emergency care, and lower utilization of some preventive services (Nicolaidis et al., 2013). Taken together with our findings regarding diagnoses of sex-steroid conditions and subclinical symptoms, it is particularly important to improve access to gynecological and cancer screening services for autistic women.

## Limitations

The current study has several limitations; most of them are related to the use of self-report questionnaires. Our recruitment methods employed a convenience-sampling framework through websites affiliated with the University of Cambridge, which may not be representative of the autistic or general populations. Our recruitment and study design likely excluded individuals without Internet access, or without the physical or cognitive abilities to complete a self-report survey; however, our recruitment strategies also allowed us to sample a large group of autistic females without intellectual disability, which is a particularly understudied population (Watkins et al., 2014). Questions pertaining to non-clinical symptoms and particularly timing of puberty relied on participants’ subjective recall of events. Thus, prospective studies of medical health are warranted. Although relying on participants’ recall may be problematic, in this case women reported on medical conditions and symptoms with significant impact to their lives, often confirmed by physicians and associated with treatment protocols and are therefore less likely to be misrepresented.

Participants’ self-report of their diagnostic status was substantiated by giving exact and objective information regarding the diagnostic procedure (e.g. who conducted the diagnoses, when was it made); a method that showed good cross-validation with psychiatric records (Daniels et al., 2012).

In addition, the findings of the study suggest that much of the association between the examined syndromes and symptoms could be mediated by sex-steroid levels, without their direct measurement. Future studies would benefit from obtaining circulating hormone samples or conducting clinically validated endocrine tests (e.g. glucose tolerance test, GnRH challenge) to further substantiate this link.

It is also worth noting that IQ was not measured in the current study, and therefore no information regarding mental capacity was included in the analysis. Providing answers to the questionnaires required reading and comprehension abilities, and for that reason, it is assumed that most of our participants did not have intellectual disabilities. Therefore, it is possible that the results do not generalize to the broader population of autistic women. Finally, the effects identified in this study are derived from a single population and would further require replication in an independent cohort to be confirmed.

## Conclusion

The current study examined the link between sex-steroid-related conditions among women with and without an autism diagnosis. It focused on four main indications of sex-steroid imbalance: medical conditions, symptoms of sex-steroid imbalance, puberty onset, and reproductive health. Many of the conditions and symptoms, such as puberty onset, menstrual length, and prediabetes symptoms, were found to be associated with having an autism diagnosis or autistic traits. The study suggests an important lifetime function of sex-steroids for autistic women and promotes our understanding of the physical symptoms and medical conditions co-occurring within autistic women. These findings have important im-plications for healthcare awareness. Prior knowledge of sex-steroid-related conditions could facilitate early diagnosis and improved prognosis for children and youths diagnosed with autism and contribute to better health outcomes in adulthood. Future studies should further investigate this association and provide a roadmap on how best to introduce these findings into healthcare protocols.

## Supplemental Material

sj-docx-1-aut-10.1177_13623613211022091 – Supplemental material for Medical symptoms and conditions in autistic women Supplemental material, sj-docx-1-aut-10.1177_13623613211022091 for Medical symptoms and conditions in autistic women by Tslil Simantov, Alexa Pohl, Alexandros Tsompanidis, Elizabeth Weir, Michael V Lombardo, Amber Ruigrok, Paula Smith, Carrie Allison, Simon Baron-Cohen and Florina Uzefovsky in Autism
