# Supplementary material for: Medical symptoms and conditions in autistic women
Source: Autism. 2021 Jun 29;26(2):373–88. doi: 10.1177/13623613211022091 (PMC8814970; doi:10.1177/13623613211022091)
Supplement: sj-docx-1-aut-10.1177_13623613211022091 – Supplemental material for Medical symptoms and conditions in autistic women [file sj-docx-1-aut-10.1177_13623613211022091.docx]

**Supplementary Materials**

**Supplementary Table 1 – Hormonal conditions**

*The relationship between testosterone and medical conditions, hormonal symptoms, puberty, and reproductive health in women.*

| Category | Syndrome/symptom | Associated with | Reference |
| --- | --- | --- | --- |
| Sex-hormone associated disorders | PCOS | Higher total testosterone levels and hyperandrogenism | (Azziz et al., 2006; Balen & Michelmore, 2002; Cesta et al., 2020; Chen, Kong, Piltonen, Gissler, & Lavebratt, 2020; Franks, 1995; Hull, 1987; Katsigianni, Karageorgiou, Lambrinoudaki, & Siristatidis, 2019; Rotem et al., 2020) |
|  | Type-1 diabetes | Normal total testosterone concentrations | (Tomar et al., 2006) |
|  |  | hyperandrogenism and PCOS | (Codner & Escobar-Morreale, 2007; Codner et al., 2006; Escobar-Morreale et al., 2000) |
|  | Type-2 diabetes | Androgenicity, as assessed by increased testosterone | (Haffner, 2000) |
|  |  | Lower total testosterone levels in comparison to type I diabetes | (Chandel, Dhindsa, Topiwala, Chaudhuri, & Dandona, 2008; Tomar et al., 2006) |
|  |  | Higher plasma levels of testosterone and estradiol in postmenopausal women | (Ding et al., 2007) |
|  |  | Testosterone deficiency, insulin resistance and type-2 diabetes | (Traish, Saad, & Guay, 2008) |
|  | Premenstrual syndrome (PMS) | Lower testosterone levels | (Hashemi et al., 2016) |
|  |  | Women with "Pure PMS" (who experience symptoms only premenstrually) showed lower testosterone concentrations than women diagnosed as having "Pre-menstrual exacerbation" (who experience symptoms throughout the cycle but with a significant exacerbation pre-menstrually) | (Ekholm, Turkmen, Hammarbäck, & Bäckström, 2014) |
|  | Epilepsy | Estrogen increases neuronal excitability and certain androgens appear to have suppressive effect on epileptic activity | (Herzog, 1999) |
|  | Tumors and growths, or ovarian, breast and uterine cancers | Higher testosterone was associated with greater breast cancer risk among postmenopausal women | (Somboonporn & Davis, 2004) |
|  |  | Postmenopausal women using estrogen and testosterone therapies have higher risk of breast cancer | (Tamimi, Hankinson, Chen, Rosner, & Colditz, 2006) |
|  |  | Increased testosterone levels among women with ovarian levels | (Mizoguchi et al., 2014; Reedy et al., 1999; Takeuchi, Ishihara, Ohbayashi, Itoh, & Maruo, 1999) |
|  |  | Elevated androgen levels are associated with greater risk of endometrial cancer | (Kaaks, Lukanova, & Kurzer, 2002) |
|  | Autoimmune disorder | Lower testosterone serum levels in patients suffering from rheumatoid arthritis (RA) | (Robinzon & Cutolo, 1999) |
|  | Hyperthyroidism | Increased plasma levels of testosterone, and elevated production rate of testosterone | (Krassas, 2000) |
|  | Hypothyroidism | Decrease in serum testosterone | (Krassas & Pontikides, 2005) |
|  | CAH | Characterized by androgen excess | (Merke & Bornstein, 2005) |
|  | Cholesterol imbalance | Testosterone levels were positively correlated with HDL cholesterol among men | (Gutai et al., 1981) |
|  |  | Androgen levels have a suppressive effect on HDL cholesterol concentration among men | (Bagatell, Knopp, Vale, Rivier, & Bremner, 1992) |
|  | Blood pressure | Animal studies about menopause suggest that the shift in the androgen-estrogen ratio after menopause and the resulting ‘imbalance’ might explain the blood pressure increase and hypertension | (Reckelhoff, 2001) |
|  |  | Among men, testosterone levels were inversely correlated with systolic and diastolic blood pressure | (Khaw & Barrett-Connor, 1988; Torkler et al., 2011) |
|  | Premenstrual syndrome | Lower testosterone levels | (Hashemi et al., 2016) |
|  |  | Women with "Pure PMS" (who experience symptoms only premenstrually) showed lower testosterone concentrations than women diagnosed as having "Pre-menstrual exacerbation" (who experience symptoms throughout the cycle but with a significant exacerbation pre-menstrually) | (Ekholm et al., 2014) |
|  | Prenatal metabolic syndrome (PNMS) | Maternal PNMS, and more specifically higher estradiol and lower sex hormone binding globulin (SHBG), was significantly associated with increased autism risk for the offspring | (Bilder et al., 2019) |
| Hormonal symptoms | Hirsutism | Hyperandrogenism | (Archer & Chang, 2004; Azziz, Carmina, & Sawaya, 2000) |
|  |  | Abnormally high levels of testosterone | (Speroff & Fritz, 2005) |
|  |  | Modest correlation among women with PCOS | (Legro et al., 2010) |
|  | Severe acne | Hyperandrogenism | (Archer & Chang, 2004) |
|  | Dysmenorrhea | Hyperandrogenism | (Caufriez, 1991) |
|  | frequent need to urinate, extreme thirst, hair loss and sudden weight loss | considered to be indicators of diabetes, which is also connected to testosterone | (Ding et al., 2007) |
| Puberty | Precocious puberty | High serum levels of testosterone are associated with precocious puberty among boys | (Carel & Léger, 2008) |
|  | Delayed puberty | Low serum levels of testosterone are associated with delayed puberty among boys | (Palmert & Dunkel, 2012) |
|  |  | Boys with delayed puberty show rapidly advancing pubertal maturation when administered testosterone | (Finkelstein et al., 1999) |
| Reproductive health | Irregular menstrual cycle | Testosterone levels were positively associated with menstrual length and menstrual irregularity | (Wei, Schmidt, Dwyer, Norman, & Venn, 2009) |
|  |  | Elevated androgen levels were associated with long menstrual cycles among Pima Indians | (Weiss et al., 1994) |
|  |  | Menstrual irregularities are associated with higher levels of circulating androgens among premenopausal women | (Van Anders & Watson, 2006) |

**Appendix A – Health and pregnancy questionnaire**

**Questions of the Health and pregnancy questionnaire included in the analysis:**

- - - 1. What was your height at age 18?
      2. What was your weight at age 18?
      3. What is your current height?
      4. What is your current weight?

**Puberty:**

10. Compared to your peers, when did your breasts first begin to develop?

11. Compared to your peers, when did you first begin to develop body hair (i.e. hair on your legs and armpits)?

12. Compared to your peers, when did your growth spurt occur?

13. Compared to your peers, when did you overall go through puberty?

**Reproductive health:**

16. During your adult years, how long was (is) your average menstrual cycle? –

17. During your adult years, how consistent was (is) the length of your menstrual cycle?-

**Hormonal disorders:**

19. Have you ever been diagnosed with any of the following conditions? Please check all that apply.

- Anovulation (failure to ovulate)
- Autoimmune Disorder
- Breast cancer/tumors/growths
- Cardiac arrhythmia/atrial fibrillation/other cardiac conditions
- Congenital adrenal hyperplasia (CAH)
- Delayed puberty
- Type I Diabetes
- Type II Diabetes
- Epilepsy
- High Blood Pressure
- High Cholesterol
- Hyperthyroidism
- Hypogonadism
- Hypothyroidism
- Ovarian cancer/tumours/growths
- Polycystic ovary syndrome (PCOS)
- Precocious puberty
- Pre-menstrual syndrome (PMS)
- Uterine cancer/tumors/growths
- Chronic Fatigue Syndrome (CFS), Myalgic Encephalomyelitis (ME) or Post-Viral Fatigue Syndrome (PVFS)

**Hormonal symptoms:**

21. Have you ever had, or do you currently have, any of the following symptoms? Please check all that apply.

- Excessive bodily or facial hair (hirsutism)
- Excessive menstrual bleeding
- Extreme thirst
- Frequent need to urinate
- Hair loss or thinning
- Severe acne
- Sudden, unexplained weight loss
- Unusually painful period

**Supplementary Table 2 – Hormonal conditions**

The 'Health and pregnancy questionnaire' contained 20 different medical conditions. During the data reduction process we examined the frequency of each condition. Conditions with none/extremely few occurrences (< 10) were removed from further analysis.

| Condition | Frequency | Valid percent |
| --- | --- | --- |
| Anovulation | 30 | 2.4 |
| Autoimmune disorder | 92 | 7.5 |
| Breast cancer/ tumours/ growths | 33 | 2.7 |
| Cardiac arrhythmia/ atrial fibrillation/other cardiac conditions | 61 | 5.0 |
| Congenital adrenal hyperplasia (CAH)* | 0 | 0 |
| Delayed puberty* | 5 | .4 |
| Type I diabetes* | 1 | .1 |
| Type II diabetes | 21 | 1.7 |
| Epilepsy | 17 | 1.4 |
| High blood pressure | 103 | 8.4 |
| High cholesterol | 85 | 6.9 |
| Hyperthyroidism | 31 | 2.5 |
| Hypogonadism * | 2 | .2 |
| Hypothyroidism | 92 | 7.5 |
| Ovarian cancer/ tumours/ growths | 23 | 1.9 |
| Polycystic ovary syndrome (PCOS) | 111 | 9.0 |
| Precocious puberty* | 6 | .5 |
| Pre-menstrual syndrome (PMS) | 210 | 17.1 |
| Uterine cancer/ tumours/ growths | 31 | 2.5 |
| Chronic Fatigue Syndrome (CFS), Myalgic Encephalomyelitis (ME) or Post-Viral Fatigue Syndrome (PVFS) | 70 | 5.7 |

*removed due to low frequency

**Supplementary Table 3 – Model summaries for hormonal conditions**

In order to reduce the number of variables, a confirmatory categorical principal component analysis (CATPCA) was conducted. Table 2 shows fit measures for the 2-, 3- and 4-factor models.

| Number of dimensions | Cronbach’s Alpha | Variance accounted for | |
| --- | --- | --- | --- |
|  |  | Total (Eigenvalue) | % of variance |
| 2 | .747 | 3.306 | 22.038 |
| 3 | .838 | 4.582 | 30.545 |
| 4 | .883 | 5.694 | 37.960 |

The 3-factor solution was chosen for further analyses. While the 4-factor solution showed slightly better fit, the fourth factor included several unrelated conditions (cardiac arrhythmia/ atrial fibrillation/other cardiac conditions; epilepsy; and ovarian cancer/tumors/growths) with a lack of theoretical support.

**Supplementary Table 4 - Factor loadings for 2 factors of hormonal conditions:**

| Condition | 1 | 2 |
| --- | --- | --- |
| Anovulation | -.113 | **.501** |
| Autoimmune Disorder | .178 | .358 |
| Breast cancer/tumours/growths | .127 | .036 |
| Cardiac arrhythmia/atrial fibrillation/other cardiac conditions | .211 | .186 |
| Type II Diabetes | **.613** | -.142 |
| Epilepsy | .012 | .198 |
| High Blood Pressure | **.690** | .019 |
| High Cholesterol | **.725** | -.090 |
| Hyperthyroidism | .349 | .193 |
| Hypothyroidism | .329 | .255 |
| Ovarian cancer/tumours/growths | -.076 | .401 |
| Polycystic ovary syndrome (PCOS) | .081 | **.529** |
| Pre-menstrual syndrome (PMS) | .174 | **.503** |
| Uterine cancer/tumours/growths | .037 | .290 |
| Chronic Fatigue Syndrome (CFS), Myalgic Encephalomyelitis (ME) or Post-Viral Fatigue Syndrome (PVFS) | .159 | .403 |

**Supplementary Table 5 - Factor loadings for 3 factors of hormonal conditions**:

| condition | 1 | 2 | 3 |
| --- | --- | --- | --- |
| Anovulation | -.100 | .028 | **.537** |
| Autoimmune Disorder | -.048 | **.513** | .138 |
| Breast cancer/tumors/growths | .231 | -.150 | .157 |
| Cardiac arrhythmia/atrial fibrillation/other cardiac conditions | .064 | .352 | .042 |
| Type II Diabetes | **.683** | .004 | -.041 |
| Epilepsy | -.011 | .078 | .182 |
| High Blood Pressure | **.667** | .217 | .031 |
| High Cholesterol | **.753** | .118 | -.028 |
| Hyperthyroidism | .083 | **.601** | -.070 |
| Hypothyroidism | .058 | **.613** | -.011 |
| Ovarian cancer/tumors/growths | -.008 | -.077 | **.492** |
| Polycystic ovary syndrome (PCOS) | .066 | .131 | **.546** |
| Pre-menstrual syndrome (PMS) | .083 | .291 | **.438** |
| Uterine cancer/tumors/growths | .217 | -.271 | **.505** |
| Chronic Fatigue Syndrome (CFS), Myalgic Encephalomyelitis (ME) or Post-Viral Fatigue Syndrome (PVFS) | .018 | .360 | .278 |

**Supplementary Table 6 - Factor loadings for 4 factors of hormonal conditions:**

| Condition | 1 | 2 | 3 | 4 |
| --- | --- | --- | --- | --- |
| Anovulation | -.097 | .039 | **.616** | -.116 |
| Autoimmune Disorder | -.033 | **.515** | .098 | .117 |
| Breast cancer/tumours/growths | .163 | -.166 | -.025 | .527 |
| Cardiac arrhythmia/atrial fibrillation/other cardiac conditions | .023 | .343 | -.176 | **.568** |
| Type II Diabetes | **.695** | -.036 | .033 | -.084 |
| Epilepsy | -.059 | .076 | .017 | **.451** |
| High Blood Pressure | **.678** | .177 | .061 | .039 |
| High Cholesterol | **.754** | .073 | -.015 | .085 |
| Hyperthyroidism | .141 | **.596** | .003 | -.185 |
| Hypothyroidism | .091 | **.608** | -.025 | .041 |
| Ovarian cancer/tumours/growths | -.072 | -.076 | .349 | **.452** |
| Polycystic ovary syndrome (PCOS) | .071 | .131 | **.621** | -.076 |
| Pre-menstrual syndrome (PMS) | .071 | .287 | **.401** | .187 |
| Uterine cancer/tumours/growths | .168 | -.281 | **.471** | .215 |
| Chronic Fatigue Syndrome (CFS), Myalgic Encephalomyelitis (ME) or Post-Viral Fatigue Syndrome (PVFS) | .030 | .361 | .285 | .036 |

**Supplementary Table 7 – Hormonal symptoms**

The 'Health and pregnancy questionnaire' assessed the existence of eight hormonal symptoms. During the data reduction process, we examined the frequency of each condition. All symptoms were retained for subsequent analyses.

| **Symptom** | **Frequency** | **Valid percent** |
| --- | --- | --- |
| Excessive bodily hair or facial hair | 113 | 9.2 |
| Excessive menstrual bleeding | 324 | 26.3 |
| Extreme thirst | 125 | 10.1 |
| Frequent need to urinate | 225 | 18.3 |
| Hair loss or thinning | 179 | 14.5 |
| Severe acne | 111 | 9.0 |
| Sudden, unexplained weight loss | 43 | 3.5 |
| Unusually painful periods | 343 | 27.8 |

**Supplementary Table 8 – Model summaries for hormonal symptoms**

In order to reduce the number of variables, a confirmatory categorical principal component analysis (CATPCA) was conducted. Table 4 shows fit measures for the 2-, 3- and 4-factor models.

| Number of dimensions | Cronbach’s Alpha | Variance accounted for | |
| --- | --- | --- | --- |
|  |  | Total (Eigenvalue) | % of variance |
| 2 | .818 | 3.519 | 43.990 |
| 3 | .889 | 4.499 | 56.239 |
| 4 | .931 | 5.397 | 67.461 |

The 3-factor solution was chosen for further analyses. While the 4-factor solution showed slightly better fit, each factor included only 2 items, which did not make an efficient data reduction.

**Supplementary Table 8 - Factor loadings for 2 factors of hormonal symptoms:**

| Symptom | 1 | 2 |
| --- | --- | --- |
| Excessive bodily or facial hair | .151 | **.415** |
| Excessive menstrual bleeding | .123 | **.785** |
| Extreme thirst | **.822** | .069 |
| Frequent need to urinate | **.772** | .115 |
| Hair loss or thinning | .453 | .278 |
| Severe acne | .057 | **.482** |
| Sudden, unexplained weight loss | **.495** | .122 |
| Unusually painful periods | .170 | **.772** |

**Supplementary Table 9 - Factor loadings for 3 factors of hormonal symptoms:**

| Symptom | 1 | 2 | 3 |
| --- | --- | --- | --- |
| Excessive bodily or facial hair | .128 | .059 | **.701** |
| Excessive menstrual bleeding | .114 | **.858** | .109 |
| Extreme thirst | **.820** | .078 | .039 |
| Frequent need to urinate | **.774** | .211 | -.091 |
| Hair loss or thinning | **.442** | .144 | .315 |
| Severe acne | .033 | .120 | **.727** |
| Sudden, unexplained weight loss | **.488** | .016 | .228 |
| Unusually painful periods | .160 | **.839** | .116 |

**Supplementary Table 9 - Factor loadings for 4 factors of hormonal symptoms:**

| Symptom | 1 | 2 | 3 | 4 |
| --- | --- | --- | --- | --- |
| Excessive bodily or facial hair | .057 | .070 | .145 | **.707** |
| Excessive menstrual bleeding | **.859** | .097 | .084 | .096 |
| Extreme thirst | .053 | **.826** | .205 | .114 |
| Frequent need to urinate | .179 | **.860** | .053 | .026 |
| Hair loss or thinning | .158 | .201 | **.553** | .198 |
| Severe acne | .111 | .048 | -.008 | **.778** |
| Sudden, unexplained weight loss | .051 | .059 | **.896** | -.019 |
| Unusually painful periods | **.840** | .131 | .119 | .099 |
